# Frequency of benign tumors after partial nephrectomy and the association between malignant tumor findings and preoperative clinical parameters

**DOI:** 10.1186/s12894-024-01543-3

**Published:** 2024-08-22

**Authors:** Veronika Lounová, Vladimír Študent, Jr., Dana Purová, Igor Hartmann, Aleš Vidlář, Vladimír Študent

**Affiliations:** 1 Department of Urology, University Hospital Olomouc Palacký University Olomouc, Olomouc, Czech Republic; 2 Olomouc University Social Health Institute, Palacky University Olomouc, Olomouc, Czech Republic

**Keywords:** Kidney tumors, Pathology, Carcinoma, renal cell, Incidence, Benign

## Abstract

**Background:**

Partial nephrectomy (PN) has become the dominant treatment modality for cT1 renal tumor lesions. Tumors suspected of malignant potential are indicated for surgery, but some are histologically classified as benign lesions after surgery. This study aims to analyze the number of benign findings after PN according to definitive histology and to evaluate whether there is an association between malignant tumor findings and individual factors.

**Methods:**

The retrospective study included 555 patients who underwent open or robotic-assisted PN for a tumor in our clinic from January 2013 to December 2020. The cohort was divided into groups according to definitive tumor histology (malignant tumors vs. benign lesions). The association of factors (age, sex, tumor size, R.E.N.A.L.) with the malignant potential of the tumor was further evaluated.

**Results:**

In total, 462 tumors were malignant (83%) and 93 benign (17%). Of the malignant tumors, 66% were clear-cell RCC (renal cell carcinoma), 12% papillary RCC, and 6% chromophobe RCC. The most common benign tumor was oncocytoma in 10% of patients, angiomyolipoma in 2%, and papillary adenoma in 1%. In univariate analysis, there was a higher risk of malignant tumor in males (OR 2.13, 95% CI 1.36–3.36, *p* = 0.001), a higher risk of malignancy in tumors larger than 20 mm (OR 2.32, 95% CI 1.43–3.74, *p* < 0.001), and a higher risk of malignancy in tumors evaluated by R.E.N.A.L. as tumors of intermediate or high complexity (OR 2.8, 95% CI 1.76–4.47, *p* < 0.001). In contrast, there was no association between older age and the risk of malignant renal tumor (*p* = 0.878).

**Conclusions:**

In this group, 17% of tumors had benign histology. Male sex, tumor size greater than 20 mm, and intermediate or high R.E.N.A.L. complexity were statistically significant predictors of malignant tumor findings.

## Introduction

Renal cell carcinoma (RCC) accounts for 2–3% of all malignancies [1]. It is also the sixth most common malignancy in men and the tenth most common in women [2].

Benign forms of kidney cancer account for approximately 15%. Due to the diagnostics, for which imaging methods (mainly CT – computed tomography) are essential, radiologists can usually distinguish these benign tumors from malignant tumors, and patients with benign lesions are only followed up. There is no clear protocol for follow-up, but clinical examination, including CT scanning, is recommended at six months, then at 12 months, and then regularly every year [3].

Nevertheless, imaging techniques are sometimes unable to distinguish small benign lesions from malignant ones. It is related to insufficiency of radiological methods in the differential diagnosis of benign-malignant tumors. Some studies deal with other new non-invasive imaging techniques that would help with preoperative diagnosis. Contrast-enhanced ultrasound (CEUS) has the potential to be a valuable alternative to CT (computed tomography) or MRI (magnetic resonance imaging) [4]. Other studies evaluate the diagnostic accuracy of 99mTc-sestamibi single-photon emission tomography in characterizing indeterminate renal masses [5]. Early data suggest that CEUS or SestaMIBI SPECT/CT is a promising option for the evaluation of renal masses, but more reliable evidence is required.

Therefore, it would be useful to investigate whether other preoperative clinical characteristics can predict benign renal tumor histology. More patients could be spared unnecessary surgery and subsequent complications [6]. When somebody has a suspicious finding, renal biopsy is indicated [7, 8].

If the benign tumor is symptomatic (most often pain or haematuria) or larger than 7 cm, this is an indication for active treatment. Active treatment includes surgery, embolization, radiofrequency ablation, or cryoablation [9]. In case of uncertainty about malignancy, biopsy or surgical treatment is indicated.

The reported rate of benign tumour after surgical treatment ranges from 7 to 33%. Studies with small numbers of patients may bias the results. When tumors are grouped by size, there is clear evidence of a higher rate of benign tumors in smaller lesions [10–16].

Recently, partial nephrectomy (PN) has become the dominant treatment for cT1 renal tumor lesions. Surgical modalities include open, laparoscopic, and robotic-assisted surgery. This study aims to analyze the number of benign findings after PN according to definitive histology and to evaluate whether there is an association between malignant tumor findings and individual factors, which could lead to a future algorithm for the probability of tumor benignity. Many patients could avoid surgery and the risk of complications associated with PN. Unnecessary surgical treatment for a benign tumor and the associated hospitalization are also an economic burden [17]. Another aim of this comparison is to point out the possibility of serious complications, even in surgery for benign tumours.

## Materials and methods

The retrospective study included patients who underwent open or robot-assisted PN for a tumor in our clinic from January 2013 to December 2020. Patients with multiple tumors were excluded from the original cohort because of the use of the R.E.N.A.L. nephrometry score and dividing to three groups according to this score.

Since most of the tumors are found incidentally, different CT protocols were applied at the time of diagnosis (different slice thicknesses, for example). Some patients also had MRIs. The lesions were often found during investigations other than aimed explicitly at kidneys (imaging during follow-up for different diseases, trauma scan, PET-CT, etc.)

A kidney lesion is classified as suspicious based on HU increase between the non-contrast and corticomedullary phases. If it is > 20 HU, the lesion is considered positive as a tumor. The increase of 15–20 is equivocal, and < 15 HU is considered benign (such as protein or hemorrhagic cysts).

Although the washout sign is not used in daily practice, it aids some information in clear cell RCC to contrast speedy washout compared to healthy parenchyma. Patterns are of limited use in kidney tumors (except for distinguishing RCC from urothelial cancers), as even most oncocytomas are hard to distinguish from RCC. Only angiomyolipoma (not fat-poor angiomyolipoma) can be successfully differentiated from RCC [18].

Every patient diagnosed with a suspicious kidney lesion is referred to a board meeting with a dedicated uro-radiologist present. There are two of them at our institution, with more than 20 years of experience. They review every CT or MRI scan presented and present their statement.

The surgical approach was chosen based on the history of abdominal surgery, patient habitus, tumor location, and surgeon and patient preferences. Open surgery was performed through a dorsal lumbotomy approach. Tumor removal was usually done using wedge resection, and hilar clamping was usually done. The surgical procedure of partial nephrectomy has been previously described in detail [19]. Robotic partial nephrectomy was done transperitoneally using DaVinci Si and Xi robotic systems. Hilar clamping was done according to the surgeons’ preference. The resection technique was either resection or enucleation and was described elsewhere [20].

The cohort was divided into groups according to the definitive histology of the tumor; the two main groups were malignant tumors and benign lesions. These were further divided into groups according to the exact histological type. For malignant tumors, these were clear-cell RCC, papillary RCC (types 1 and 2 combined), chromophobe RCC, and a group termed “other”, which included other malignant tumors, most commonly those with a sarcomatoid component. There were subgroups for benign tumors - oncocytoma, angiomyolipoma, papillary adenoma, and also an “other” group, which included, e.g., cystic nephroma, cyst without malignant potential.

Both groups were compared in terms of perioperative data. The tumors were assessed according to the R.E.N.A.L. nephrometric score and divided into three groups according to complexity. The first group included tumors with low complexity (scores 4–6), the second group included tumors with intermediate complexity (scores 7–9), and the last group included tumors with high complexity (scores 10–12). Because of the smaller number of patients in the intermediate and high complexity groups, these two groups were combined into one. The R.E.N.A.L. nephrometry scores the tumor according to size, distance from the collecting system, location relative to the polar lines, whether it is exophytic or endophytic, and whether it is more likely to be ventrally or dorsally located [21].

Patients were also sorted into three groups according to age - the first group consisted of patients under 60 years of age, the second group consisted of patients 60–70 years of age, and the third group consisted of patients over 70. There was a further division into male and female and two groups according to tumor size (20 mm and smaller and the second group - larger than 20 mm).

Tumors were divided into malignant and benign, and the following characteristics were compared within these two groups: patient age at the time of surgery, Charlson comorbidity index, tumor size, length of surgery, blood loss, warm ischemia time, and glomerular filtration rate before surgery, immediately after surgery, and at six months after surgery. Particular attention was paid to complications, which were divided into five grades according to the Clavien-Dindo classification and further according to the nature of the complication into bleeding, infectious, urinoma, and other complications. The aim of this comparison is to point out the possibility of serious complications, even in surgery for benign tumours.

Tables 1 and 2 show baseline characteristics analyzed by nonparametric’s tests. Continuous variables are presented as medians and ranges, categorical values as absolute frequencies, and relative frequencies in %. To compare groups, in the case of continuous variables the Mann-Whitney nonparametric test was used. The distribution of categorical variables was evaluated using the Fisher test or Pearson chi-squared test. A univariate logistic regression model was built for each factor to identify those factors associated with benign histology. The odds ratio (OR) with a 95% confidence interval (CI) and p-values is summarized in Table 3. All analysis was performed using Statistica V.13.4.0.14 (Tibco Software Inc., VA, USA) and R software, version 4.1.0 (www.r-project.org). The level of significance was set at 5% for all statistical tests.

## Results

In total, partial nephrectomy was performed in 555 patients. Four hundred sixty-two tumors were malignant (83%) and ninety-three benign (17%). Of the malignant tumors, 66% were found to be clear cell renal cell carcinoma, 12% papillary renal cell carcinoma, and 5% chromophobe renal cell carcinoma. The most common benign tumor was oncocytoma in 10% of patients, angiomyolipoma in 2%, and papillary adenoma in 1%.

There were no differences between the groups in age (*p* = 0.480) and Charlson comorbidity index (*p* = 0.456). On average, the surgery for benign tumors was shorter compared to partial nephrectomy done in malignant tumors (90.66 vs. 97.31 min, *p* = 0.018). The median estimated blood loss (EBL) was lower in benign tumors (100 vs. 200 ml, *p* < 0.001). The benign tumor’s median size was smaller than the malign ones (25 vs. 30 mm, *p* = 0.007). Functional results showed shorter warm ischemia time in benign tumors (*p* < 0.001). There was, however, no difference in eGFR rates before (*p* = 0.459), after the surgery (*p* = 0.822), and six months post-surgically (*p* = 0.805) (Table 1).

**Table 1:** Comparison of malignant and benign tumours in terms of various variables

	Malignant tumors					Benign tumors					*p*-value
	*n*	average	median	min	max	*n*	average	median	min	max	
age	462	62.96	64.00	30.00	85.00	93	63.76	66.00	38.00	82.00	0.480
Charlson	462	4.74	5.00	0.00	11.00	93	4.58	4.00	2.00	10.00	0.456
length (min)	462	97.31	95.00	29.00	180.00	93	90.66	90.00	29.00	223.00	0.018
Estimated blood loss (ml)	459	243.74	200.00	0.00	2000.00	88	161.53	100.00	0.00	1200.00	< 0.001
ischemia (min)	459	10.47	11.00	0.00	45.00	82	6.77	0.00	0.00	26.00	< 0.001
GF_before (ml/s/1,73m2)	338	1.16	1.19	0.14	1.50	58	1.13	1.20	0.34	1.50	0.459
GF_after (ml/s/1,73m2)	418	1.09	1.13	0.12	1.50	84	1.11	1.13	0.32	1.50	0.822
GF_after 6 m (ml/s/1,73m2)	184	1.09	1.14	0.13	1.50	24	1.08	1.12	0.27	1.50	0.805
size (mm)	462	31.60	30.00	8.00	102.00	93	30.45	25.00	5.00	160.00	0.007

Table 2 shows the stratification of malignant and benign tumors into groups according to age (< 60, 60–70, > 70), gender (male, female), tumor size (≤ 20 mm and > 20 mm), R.E.N.A.L. nephrometric score (low, medium, high) and type of operation (robot, open). All categories, except age, were evaluated as statistically significant. Men generally had a higher incidence of benign tumors (51.61%) and malignant (69.48%), *p* = 0.001. Furthermore, significantly more tumors larger than 20 mm were operated on, 63.44% benign and 80.08% malignant (*p* < 0.001). According to R.E.N.A.L. only 34.41% of benign tumors were evaluated as intermediate + high complexity, whereas for malignant tumors, it was 59.52% (*p* < 0.001). Robotic surgery was performed in 64.52% of benign tumors, compared to 45.89% of all malignant tumors (*p* < 0.001).

**Table 2:** Malignant and benign tumors according to groups, basic characteristics

*n* = 555		tumor				p-value
		benign = 93		malignant *n* = 462		
		n	%	n	%	
age	< 60	28	30.11%	147	31.82%	0.878
	60–70	36	38.71%	193	41.77%	
	> 70	29	31.18%	122	26.41%	
sex	M	48	51.61%	321	69.48%	0.001
	F	45	48.39%	141	30.52%	
tumor size	<=20 mm	34	36.56%	92	19.91%	< 0.001
	> 20 mm	59	63.44%	370	80.08%	
RENAL	low	61	65.59%	187	40.48%	< 0.001
	med + high	32	34.41%	275	59.52%	
surgery	robot	60	64.52%	212	45.89%	0.001
	open	33	35.48%	250	54.11	

In univariate, analysis (Table 3) there was a higher risk of malignant tumor finding in males (OR 2.13, 95% CI 1.36–3.36, *p* = 0.001), a higher risk of malignancy in tumors larger than 20 mm (OR 2.32, 95% CI 1.43–3.74, *p* < 0.001), and a higher risk of malignancy in tumors evaluated by R.E.N.A.L. as tumors of intermediate or high complexity (OR 2.8, 95% CI 1.76–4.47, *p* < 0.001). In contrast, there was no association between older age and the risk of malignant renal tumor (*p* = 0.889).

**Table 3:** Association of specific factors with tumour malignancy

covariate	malignant x benign					
	unadjusted			full model		
	OR	95%CI	*p*-value	OR	95%CI	*p*-value
age:<60	0.98	0.57–1.68	0.645	0.9	0.51–1.57	0.889
age:60–70	Reference			reference		
age:>70	0.78	0.46–1.35		0.88	0.5–1.55	
sex: M vs. F	2.13	1.36–3.36	0.001	2.22	1.38–3.56	0.001
tumor size:>20 vs. <=20	2.32	1.43–3.74	< 0.001	1.92	1.16–3.19	0.013
renal: m + h vs. low	2.8	1.76–4.47	< 0.001	2.54	1.56–4.12	< 0.001

Table 4 deals with complications of surgery for malignant and benign tumors. Of the 474 patients evaluated for complications with malignant tumor and 89 patients with benign tumor, 59 malignant (12.45%) and 10 benign (11.24%) patients experienced some form of complication, *p* = 0.749.

After dividing the complications according to the Clavien-Dindo classification, the complications were as follows: Clavien-Dindo 1 + 2–35 malignant (7.38%) and seven benign (7.87%). Clavien-Dindo 3a − 16 malignant (3.38%) and one benign (1.12%), Clavien-Dindo 3b − 7 malignant (1.48%) and one benign (1.12%), Clavien-Dindo 4 + 5 - one malignant (0.21%) and one benign (1.12%) with p-value = 0.520.

Further division of complications is possible into bleeding, infectious, urinary leakage, and other complications. Bleeding occurred in 27 (45.76%) patients with malignant tumors and 5 (50%) with benign tumors, and infectious complications in 16 (27.12%) with malignant and none with benign. Urinoma occurred in 10 (16.95%) patients with malignant tumor and 1 (10%) with benign tumor. Other rare complications (e.g., pulmonary embolism, ileus) occurred in 6 (10.17%) malignant and 4 (40%) benign.

**Table 4:** Comparison of complications in patients with malignant and benign tumours

		malignant tumor			
		**yes**	**no**	*Totals*	p-values
complications	**0**	415	79	494	0.749
	*Column %*	87.55%	88.76%		
	*Row %*	84.01%	15.99%		
	**1**	59	10	69	
	*Column %*	12.45%	11.24%		
	*Row %*	85.51%	14.49%		
Clavien- Dindo	**0**	415	79	494	0.520
	*Column %*	87.55%	88.76%		
	*Row %*	84.01%	15.99%		
	**1 + 2**	35	7	42	
	*Column %*	7.38%	7.87%		
	*Row %*	83.33%	16.67%		
	**3a**	16	1	17	
	*Column %*	3.38%	1.12%		
	*Row %*	94.12%	5.88%		
	**3b**	7	1	8	
	*Column %*	1.48%	1.12%		
	*Row %*	87.50%	12.50%		
	**4 + 5**	1	1	2	
	*Column %*	0.21%	1.12%		
	*Row %*	50.00%	50.00%		
complications	**urinoma**	10	1	11	0.043
	*Column %*	16.95%	10.00%		
	*Row %*	90.91%	9.09%		
	**bleeding**	27	5	32	
	*Column %*	45.76%	50.00%		
	*Row %*	84.38%	15.63%		
	**infectious**	16	0	16	
	*Column %*	27.12%	0.00%		
	*Row %*	100.00%	0.00%		
	**other**	6	4	10	
	*Column %*	10.17%	40.00%		
	*Row %*	60.00%	40.00%		

## Discussion

Resection of the kidney is a good standard for the treatment of cT1a renal tumors, which is reflected in American and European recommendations. Renal tumors are often found incidentally by imaging. Improvements in preoperative imaging techniques have led to more frequent detection of small tumors, but these imaging techniques are sometimes unable to distinguish primarily these small benign lesions from malignant ones. As with any surgical procedure, there is potential morbidity associated with renal resection, and thus, active surveillance is preferable to surgical treatment in some patients with a higher likelihood of benign tumor [6].

The aim of this study was to analyze the number of benign findings after renal resection according to definitive histology and to evaluate whether there is an association between malignant tumor findings and individual factors, as in previously published studies.

The reported rate of benign tumor after surgical treatment is 7–33% in the published literature. However, this figure is biased by studies with low numbers of patients [6].

Therefore, Baumann et al. [6] worked with a large series of 916 patients, demonstrating an incidence of benign tumors in 14.1% of the total.

In our study, the incidence of benign tumors was 17%.

In the 2013 systematic review, Corcoran et al. [22] identified 26 representative studies that included a cohort of 27,272 patients.

The incidence of benign tumors ranged from 7 to 33%. According to this review, benign renal tumors represent approximately 15% of surgically resected renal masses identified and are more common among small lesions less than 4 cm (T1a) [22].

Another study in Korea investigated the incidence and predictive factors of benign renal lesions undergoing surgery for presumed renal cell carcinoma on preoperative imaging. The study involved 1598 patients with unilateral, non-metastatic, and non-familiar renal lesions. Of the 1598 renal lesions, 114 (7.1%) were benign, including angiomyolipoma in 47 (2.9%), oncocytoma in 23 (1.4%), and complicated cysts in 18 (1.1%) patients. On univariate analysis, the proportion of benign lesions was significantly higher in women and in patients with smaller tumors. The proportion of benign versus malignant lesions decreased significantly with increasing tumor size. Furthermore, the majority of cystic renal masses were classified as benign. What appears to be an interesting finding and not included in our study is the information that macroscopic hematuria was not present in patients with benign tumors. Female gender, cystic renal lesions, and smaller tumor size are independent predictors of benign histologic features, according to this study [23].

This study, however, includes all removed tumors, i.e., from resections and nephrectomies. The absolute number of benign lesions is more than half lower, but this demonstrates the correlation of malignancy with tumor size. Tumor size is also related to the R.E.N.A.L. nephrometric score, which was not considered in the Korean study. Otherwise, the study also showed a higher risk of malignancy in the male gender. Another interesting finding is the higher incidence of angiomyolipoma than oncocytoma, in contrast to our study.

Mauermann et al. [24] studied 143 benign renal tumors treated surgically. Oncocytoma was the most common benign tumor (44%), and angiomyolipoma was found in 37% of patients. Oncocytoma (10%) was also the most common in our study, but angiomyolipoma (2%) was five times less common.

Similar to Nandanan et al. [25], we also showed an association of malignancy with the male sex and high R.E.N.A.L. score. However, unlike our results, they found an association of malignancy with higher BMI, tumors extending into the renal hilus, and tumors classified as cT3a. In addition to the association of malignancy with higher BMI, higher R.E.N.A.L. score, and tumor size, Bauman et al. [6] showed an association of benign lesions with lower creatinine value. Patients with benign tumors in this study had significantly shorter operative time and less blood loss. The authors did not find any difference in the rate of complications and the need for blood transfusion. In our study, there was no statistically significant difference in glomerular filtration rate between malignant and benign tumors. However, as in this study, mean operative time was lower in benign tumors, as was blood loss [6].

Srougi et al. [26] showed a significantly higher incidence of benign lesions in tumors smaller than 3 cm.

In their study, Schachter et al. [27] demonstrated, in addition to the association of tumor size with malignancy, another important fact directly in histopathological classification. There was a higher incidence of benign and papillary tumors and a lower incidence of clear cell renal cell carcinoma in tumors smaller than 4 cm. Given the differences in the biological behavior of the different histopathological subtypes, these data are very important.

In a cohort of 815 patients with tumors smaller than 7 cm, Snyder et al. [16] did not show an association of tumor size with malignancy, but in this study women were almost twice as likely to have a benign lesion. Thus, the total number of benign tumors operated based on suspicious preoperative imaging in this cohort was 16.4%.

In case of uncertainty about malignancy, biopsy or surgical treatment is indicated. Recently, renal biopsy has been performed more frequently. This is not only because of the increasing incidence of renal cell carcinoma and benign renal tumors, but also because of the recognition that a soft tissue lesion of the kidney with contrast agent detection should not automatically be classified as renal cell carcinoma and operated on.

Alternative small renal lesion management methods, like radiofrequency ablation or active surveillance, have been used more frequently. In all these cases, histological verification is mandatory [28, 29].

Kidney biopsy has not been much preferred in the past because of the risk of seeding into the tract, error during sampling, inability to make a pathological diagnosis in some cases, and the risk of complications such as bleeding. With the improvement of percutaneous imaging techniques and the cumulative experience reported in the literature, contemporary data support performing a biopsy. There is a minimal risk of tumor seeding and histological verification can be made in up to 99% of cases [28, 29]. Given these favorable results and in view of the elderly population in whom small suspicious renal lesions are usually discovered incidentally, a biopsy may play an increasingly important role in the near future [30].

There have also been significant advances in immunohistochemical analysis that can support treatment decision-making with renal lesion biopsy. Historically, differentiating epithelioid angiomyolipoma from sarcomatoid renal cell carcinoma on pathology may have been problematic, but current immunohistochemical evaluation allows the diagnosis to be made with high accuracy. However, the diagnosis of chromophobe carcinoma or oncocytoma remains a problem in pathology due to overlapping features [30, 31].

Several studies have shown that a significant percentage of all resected solid renal tumors were benign and, therefore, unnecessarily operated on. However, if there are clear signs of malignancy on contrast imaging, radical surgery should be performed as soon as possible, in which case a biopsy would mean unnecessary delay. When there are clear signs of benignity, a renal biopsy should not be performed, as the patient would be unnecessarily exposed to the potential complications associated with renal biopsy. Diagnosing a benign lesion can be established with high accuracy using imaging methods [32].

According to our study, complications occur similarly in surgery for malignant and benign tumors. Any complication occurred in 10 (11.2%) of benign tumors. More importantly, there were 3 (3.4%) cases of severe complications (Clavien ≥ 3) in this group. Limiting unnecessary surgeries for benign lesions seems to be of high importance.

The major limitation of our study is the retrospective design. Because the evaluation was only for partial nephrectomies, we do not know how many patients with benign lesions underwent radical nephrectomy or how many patients with small benign lesions were under active surveillance either with progression and subsequent surgery or are still being followed without progression or died from other causes.

Patients with comorbidities may also be on watchful waiting or undergo radiofrequency ablation or cryoablation. This information is also not available in our study.

We hypothesize that if radical nephrectomies were included in the cohort, the percentage of benign lesions would be lower, which is evidence of the association of malignancy with tumor size and R.E.N.A.L. nephrometric score.

Another limitation of the study may be the tumor size - for this value, we used the final pathological diameter of the tumor instead of the diameter measured radiologically. Schlomer et al. [33] reported that preoperative CT might slightly overestimate tumor size. On the other hand, the time delay between imaging and pathological examination of the removed tumor may also vary.

High-quality, non-invasive preoperative differentiation of suspicious renal lesions would be a significant advance that could allow physicians greater diagnostic certainty and guide patient management through better risk stratification. Based on the association of certain factors with malignancy, a certain percentage of suspicious benign tumors could only be followed up, and the adverse effects of radical treatment could be avoided and delayed. For these patients, the follow-up would be proposed, which would lead to further studies and comparisons as to whether this so-called deferred treatment makes sense. Patients with anesthetic risk would particularly benefit from this procedure.

Significant progress has been made recently regarding treatment options for renal tumors, especially cT1a tumors, where patients can be offered other treatment options besides surgery - radiofrequency ablation, cryoablation, embolization, or active surveillance - based on other facts. In particular, patients could benefit from active surveillance if the factors associated with tumor malignancy are taken into account. However, each patient must be treated in a highly individual manner, also taking into account age, comorbidities, and personal attitude.

## Conclusions

According to our study, 17% of tumors with definite benign histology were removed during PN. Male gender, tumor size greater than 20 mm, and moderate or high R.E.N.A.L. complexity were statistically significant predictors of malignant tumor findings. In contrast, the association of malignancy related to increasing or decreasing age was not demonstrated.

## Data Availability

The datasets used and analysed during the current study are available from the corresponding author on reasonable request.
